# Localized exciton emission from monolayer WS_2_ nanoribbon at cryogenic temperature

**DOI:** 10.1515/nanoph-2024-0583

**Published:** 2025-01-07

**Authors:** Gang Qiang, Ashley P. Saunders, Cong T. Trinh, Na Liu, Andrew C. Jones, Fang Liu, Han Htoon

**Affiliations:** Center for Integrated Nanotechnologies, Materials Physics and Applications Division, 5112 Los Alamos National Laboratory, Los Alamos, NM 87545, USA; Department of Chemistry, Stanford University, Stanford, CA 94305, USA

**Keywords:** WS_2_ nanoribbon, localized exciton emission, low-temperature photoluminescence

## Abstract

We conducted low-temperature photoluminescence (PL) spectroscopy experiments on individual WS_2_ and MoSe_2_ nanoribbons prepared by gold-assisted exfoliation from the slanted surface of bulk crystals with a vicinal and stepwise pattern. The nanoribbons are predominantly monolayer and have widths varying from hundreds of nanometers down to tens of nanometers. Most MoSe_2_ NRs display an emission profile similar to 2D excitons of MoSe_2_ monolayers. In contrast, WS_2_ nanoribbons are characterized with sharp emission peaks that can be attributed to the emission from localized excitons or trions. Moreover a broad low energy emission peak can be also observed from some of the WS_2_ nanoribbons, which originates from bilayer regions. In this manuscript, we analyze spectral diffusion behavior along with pump power and temperature dependence of the localized exciton emission peaks, shedding light on potential of TMDC nanoribbons in sensing and opto-electronic applications.

## 1 Introduction

Two-dimensional (2D) atomically thin transition metal dichalcogenides (TMDCs) host rich quantum phases in monolayer, bilayer, and multilayer motifs with reduced dielectric screening, in addition to enhanced excitonic and many body effects in the nanoconfined structures [1], [2], [3], [4]. They have broad applications in a variety of fields such as transistors, sensors, quantum information and processing devices [5], [6], [7], [8]. If 2D monolayers can be further confined in an additional dimension, i.e., as a quasi-one-dimensional structure such as 1D nanoribbons (NRs), new physical and chemical properties arise [9], [10]. As a result, atomically-thin NRs have emerged as new candidates for exotic quantum phenomena beyond those achieved in their bulk and monolayer counterparts.

In 2D TMDCs, the defects, doping and strain are significant tools to manipulate electrical, optical and magnetic properties, leading to new opportunities in material design. For example, the presence of local defects or strain in TMDC monolayers and heterostructures leads to emergence of quantum emitters [8], [11], [12], [13], [14], [15], [16], [17], [18], [19], [20], [21], [22], [23], [24], [25], [26], [27], [28], [29], [30], [31], an essential building block for optical quantum computing and communication [32], [33]. Doping TMDC monolayers with atoms such as V, Mn, Co, or Cr leads to tunable magnetic properties [34]. Compared with their 2D counterparts, NRs act as a 1D confined system which can be more susceptible to local strain, defects, and edge doping effects. This opens up a broader parameter space for modifying optical and electronic properties on demand. Extensive theoretical investigations have been carried out on the electronic and magnetic properties of TMDC NRs [35], [36], [37], [38], [39] as well as their tunability via strain [40], [41], [42], [43], electric field [44], transition metal doping [45], defect states [46], [47], and on their thermoelectric properties [48], [49], [50], [51], magnetoresistance properties [52], and spin-related properties [53], [54], etc. For instance, first-principles density functional theory calculations by López-Urías et al. [38] discovered an optical polarization anisotropy enhanced well-defined absorption peak in WS_2_ NRs, with polarization along the NR axis. Under bending conditions, WSe_2_ NRs exhibit an enhanced spin–orbit coupling effect and a spatially varying spin-polarization in bands around the Fermi level, with a large tunability on the optical absorption spectrum within the near infrared region [54], [55]. In MoS_2_ NRs [56], they also assessed large tunability of band gaps and optical absorptions for NRs of different widths under different bending curvatures.

However, despite the extensive theoretical studies, experimental optical probing of the local electronic properties, particularly demonstrating the effect of doping or strain on TMDCs NRs and their potential for hosting quantum emitters, is rarely reported in the literature, especially at cryogenic temperature. The experimental progresses in nanoribbons have been hindered by the lack of available high-quality samples. Few types of NRs can be accessed through synthesis [57]. On the other hand, in NRs created through top-down, lithographic processing of monolayers, the structural and chemical integrity of the crystal edges are often affected by the writing, resist patterning, and lift-off stages. Recently, a gold-assisted technique has been developed to exfoliate parallel-aligned single crystalline nanoribbons directly from the bulk Van der Waals crystals (vdW) [58]. It is a universal top-down exfoliation technique, akin to scotch tape exfoliation in producing 2D monolayers, with a broad application to a variety of 2D NR species. In these structures, the edges of nanoribbons are freshly torn from the vdW single crystal lattices, offering an excellent platform to investigate the effect of doping and strain in the NR structure.

In this work, we exfoliated WS_2_ and MoSe_2_ NRs using this gold-assisted technique [58], and spatially resolved their photoluminescence (PL) properties systematically at cryogenic temperatures. While most MoSe_2_ NRs are characterized with 2D exciton emission similar to the monolayers, WS_2_ NRs display spectrally sharp PL peaks distributed at different positions along the length of the NRs and sometimes a low-energy broad band emission. We attributed the former to excitons or trions localized at traps and the latter to bilayer emission. Spectral diffusion behavior along with pump power and temperature dependence of the localized excitonic emission peaks are also analyzed.

## 2 Sample synthesis

Our samples are prepared using the gold-assisted exfoliation technique in Ref. [58]. In brief, we directly exfoliate nanoribbons from the as-grown slanted surface of a bulk vdW crystal at a non-zero polar cut angle, with a vicinal and stepwise pattern. To initiate the exfoliation, a 100 nm thick layer of gold (Au) is evaporated onto the crystal’s surface, followed by spin-coating with a polyvinylpyrrolidone (PVP) layer to prevent contamination. When the gold layer is lifted with a rigid and flat thermal release tape (TRT), it effectively exfoliates monolayer steps from the bulk crystal surface in the form of aligned nanoribbons. The resulting assembly, consisting of TRT/PVP/Au/nanoribbons, is transferred onto a destination SiO_2_/Si substrate. The TRT is removed through heating, and PVP is dissolved in water, leaving behind a clean Au layer. This Au layer is then etched using KI/I^−^ solution. After cleaning with water, flat and parallel aligned nanoribbons are obtained on the SiO_2_/Si substrate. NRs are characterized to be predominantly monolayer and single-crystalline, while they exhibit high aspect ratios and widths varying from hundreds of nanometers down to tens of nanometers.

## 3 Experimental results

**Figure 1:**
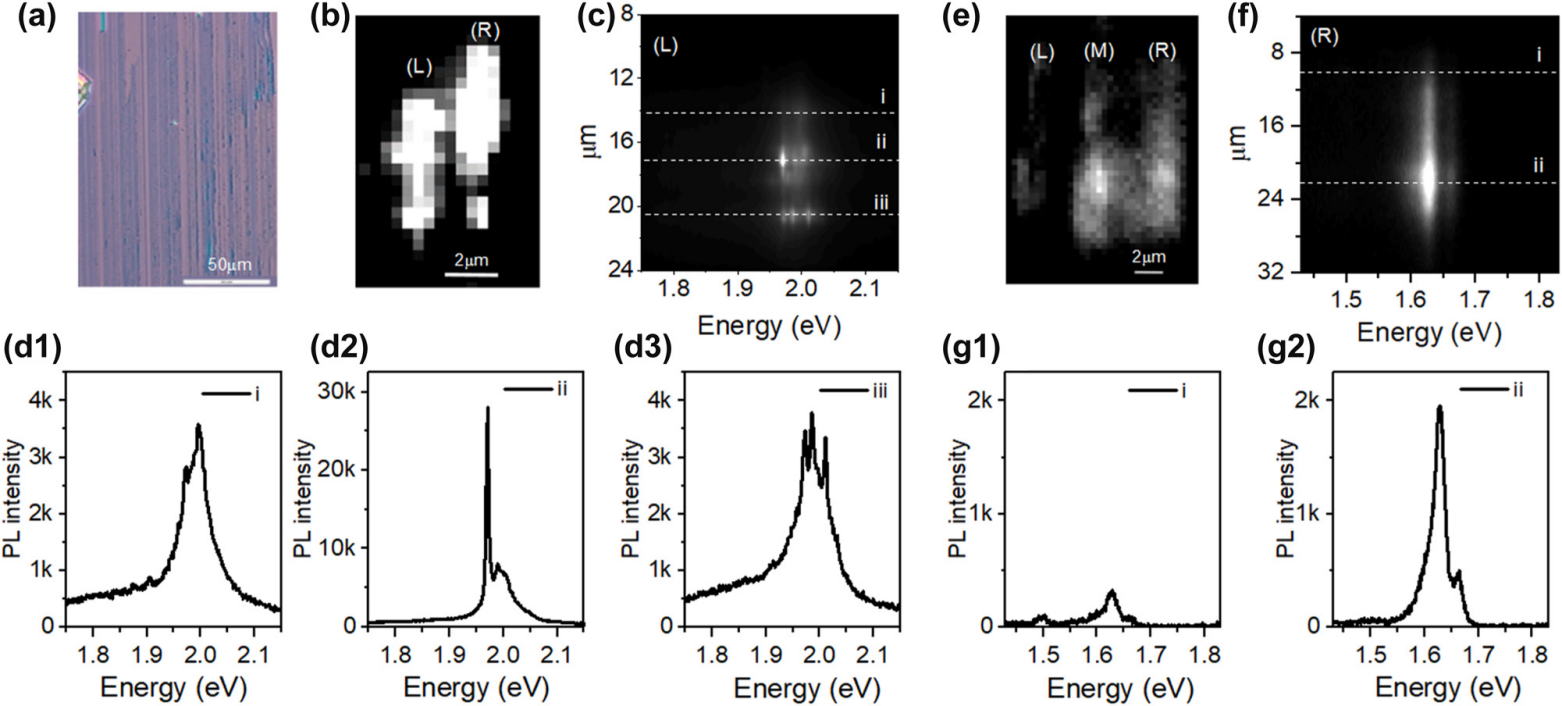
Optical properties of WS__2__ and MoSe__2__ nanoribbons at low temperature. (a) Optical image of WS_2_ NRs, the violet background corresponds to the Si/SiO_2_ substrate, the blue strips are the nanoribbons. (b) Wide-field photoluminescence (PL) image of WS_2_ NRs, two are covered within the laser spot and marked as *L* and *R*, respectively. (c) PL signal of *L* ribbon dispersed on CCD chip, and (d1)–(d3) corresponding PL spectra from specific pixel positions, namely i – iii in (c) as indicated by the white dashed lines. (e) Wide-field PL image of MoSe_2_ NRs, three NRs are resolved which are labeled as *L*, *M* and *R*. (f) PL signal of *R* ribbon dispersed on CCD chip, and (g1) and (g2) corresponding PL spectra from pixel position i – ii in (f) as guided by the white dashed lines. Experiments are done at *T* = 4.4 K.

Figure 1(a) shows the optical image of WS_2_ NRs prepared on SiO_2_/Si substrate. PL intensity profiles mapped for two NRs, marked as *L* and *R*, under 532 nm CW excitation are shown in Figure 1(b). The PL intensity from WS_2_ demonstrates bright spots at multiple locations on the NR. To characterize the spatial inhomogeneity of local PL emissions from individual NRs, we use a slit set to the image plane to confine the PL measurement to the target NR, as shown in Figure 1(c). Across multiple locations along each NR, our results demonstrate a high degree of variation in the PL intensity and shape of the emission spectra. For example, certain spots display a similar emission profile to isolated monolayer WS_2_ (Figure 1(d1)), while other spots demonstrate very sharp emission peaks at energies near the excitonic emission energy of monolayer WS_2_ (Figure 1(d2)) [59], [60]. We also carried out the same measurements on MoSe_2_ NRs, the results are demonstrated in Figure 1(e) and (f), (g1) and (g2). Similar to the WS_2_ nanoribbons, we also identified sharp emission lines for certain locations on MoSe_2_ NR samples, as indicated in Figure S7 in the Supplementary Materials. However, for the majority of MoSe_2_ NRs, the emission profiles are very similar to those of isolated MoSe_2_ monolayers, and they are pretty consistent between different nanoribbons and also along different locations of one nanoribbon.

**Figure 2:**
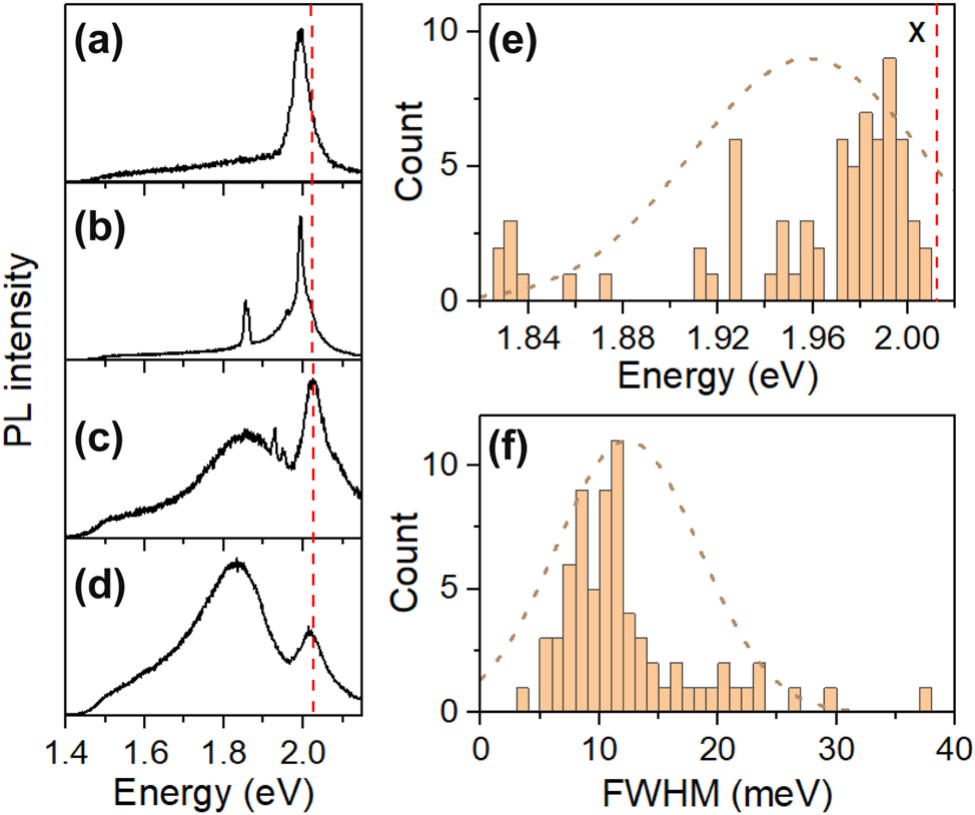
Statistics for sharp emission lines. (a)–(d) PL spectra measured on different WS_2_ NRs. Statistics of sharp emission line (e) energy and (f) full-width at half maximum (FWHM). The dashed vertical red line marks the exciton (X) emission energy of monolayer WS_2_. All data are collected at *T* = 4.4 K.

Figure 2(a)–(d) present four representative PL spectra from different WS^2^ NRs. The prominent peak around ∼2 eV corresponds to the PL emission of WS^2^ monolayer, including exciton emission and trion emission that occur approximately 20 meV apart in energy [59], [60]. The relative intensities of exciton and trion emissions vary across different NRs and even at different positions along a single NR, reflecting broad variations in local doping densities. Overall, the width and position of the exciton/trion peak are consistent with those observed in WS^2^ monolayers exfoliated using gold tape, indicating comparable crystal quality between the NR and monolayers produced by similar techniques. In some NRs, an additional emission feature appears at a lower energy, around ∼1.8 eV. This feature, based on its energy and peak shape, is attributed to bilayer regions, where the emission arises from phonon-assisted exciton annihilation [61].

In some NRs and specific locations along them, additional sharp peaks emerge at energies between the exciton/trion emissions of bilayers and monolayers, as shown in Figure 2(b) and (c). The statistics of their positions and the full width at half maximum (FWHM) of the main sharp emission lines are presented in Figure 2(e) and (f). These PL features likely originate from deep trap states in the system, such as localized defects or lattice dislocations. Similar features have been observed in monolayers under localized strain, which have the potential to function as single-photon emitters in 2D systems [19], [20]. The width of these peaks varies considerably, though most are centered around 10 meV, with the narrowest sharp emission peak exhibiting an FWHM of 3.8 meV, reflecting the diverse local environments.

**Figure 3:**
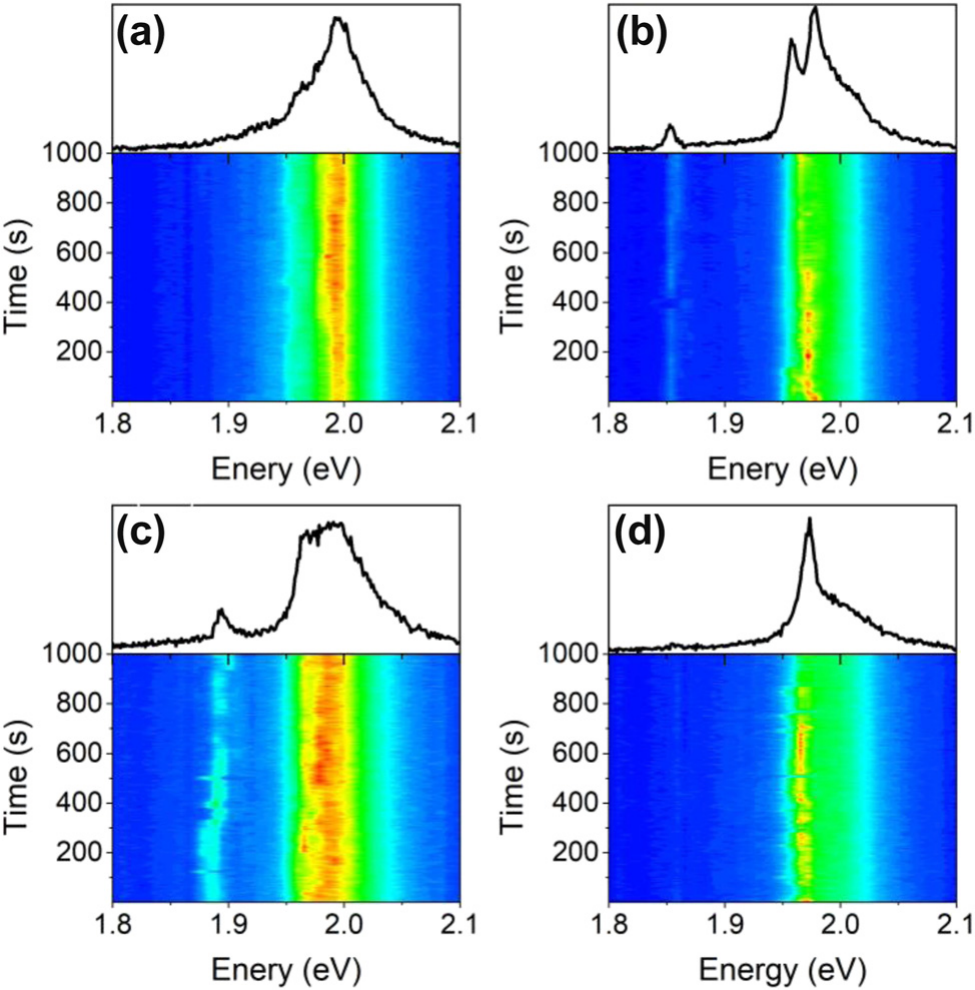
Time-dependent changes of sharp emission lines for four different locations on different nanoribbons. (Upper) PL spectra, (lower) corresponding time traces. Measurements taken at *T* = 4.4 K.

We further evaluated the evolution and stability of representative PL spectroscopic signatures over time. A few instances are shown in Figure 3. The upper part of Figure 3(a)–(d) corresponds to PL spectra, and the lower part represents their evolvements in the time-domain. Spectral wandering has been reported before for localized emitters in ML WS_2_ driven by both optical and electrical excitations and commonly attributed to fluctuation of dielectric environment [18], [24]. However, not all sharp emission lines in WS_2_ NRs show clear spectral wandering. For example, the sharp peak around 1.892 eV in Figure 3(c) fluctuates over time within a range about 9.5 meV, while the peak at 1.825 eV in Figure 3(b) and the peak at 1.973 eV in Figure 3(d) show no observable spectral wandering. In addition, all sharp emissions demonstrate clear blinking behavior, which is due to the change of dielectric environment around the emitters [27]. We also characterized the sharp emissions lines from MoSe_2_ NRs, and they also show intensity blinking and spectra wandering as shown in Figure S7.

**Figure 4:**
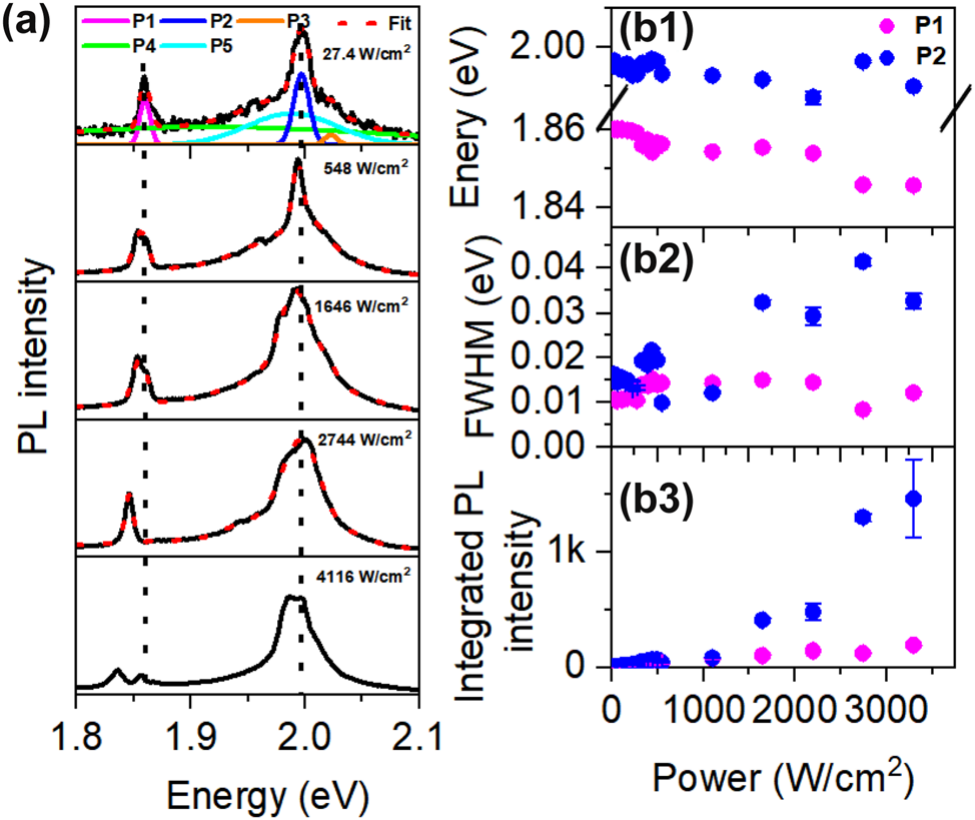
Laser excitation power dependent PL measured on a selected WS_2_ NR. (a) PL spectra measured under different excitation power. The dotted red lines are fits to five Gaussian peaks (example shown in the top panel). Dashed vertical black lines are guides for eye. (b1) – (b3) Excitation power dependence of the peak position, full-width at half maximum (FWHM), integrated PL intensity for sharp emission peaks, P1 and P2. Experiments are done at *T* = 4.4 K.

Excitation power dependence of the PL was studied on a select WS_2_ NR, the signal dispersion on the CCD chip can be found in Figure S1. At relatively low excitation power, two sharp emission peaks appear in the spectra, one is very close to the *K* point exciton peak, and the other one is away on the lower energy side in the energy range of indirect excitonic emission from bilayers. This peak however displays linewidth (∼10 meV) significantly narrower than the linewidth (∼220 meV) of bilayer emission (Figure 2c and d), and its intensity is comparable to the direct exciton emission of monolayer. Based on these facts, we conclude that this peak originated from a deep trap state of direct monolayer exciton rather than the indirect bilayer exciton emission. Figure 4(a) shows the spectra measured under different excitation powers. To analyze the data, we fit each spectrum with five Gaussian functions, and extract the peak positions, width and integrated intensity for the two major peaks (P1 and P2) at different excitation density, as shown in Figure 4(b1)–(b3). Relevant parameters for P3–P5 can be found in Figure S3. From the fitting, we see that the energy position of P2 (blue) remains almost constant with increasing laser excitation power. However, for P1 (magenta), it decreases by 14 meV from 1.860 eV under 27.4 W/cm^2^ to 1.846 eV under 3293 W/cm^2^. The width of P1 rises from about 15 meV to 40 meV in the current power range, while the width of P2 remains around 12 meV. Note that under laser excitation powers >4,000 W/cm^2^, the shape of the PL spectrum changes a lot, as indicated in Figure S2. The integrated PL intensity for P1 increases linearly with increasing excitation power, while the intensity of P2 begins by showing linear dependence at lower excitation power (<1,000 W/cm^2^), then increases nonlinearly at higher power.

**Figure 5:**
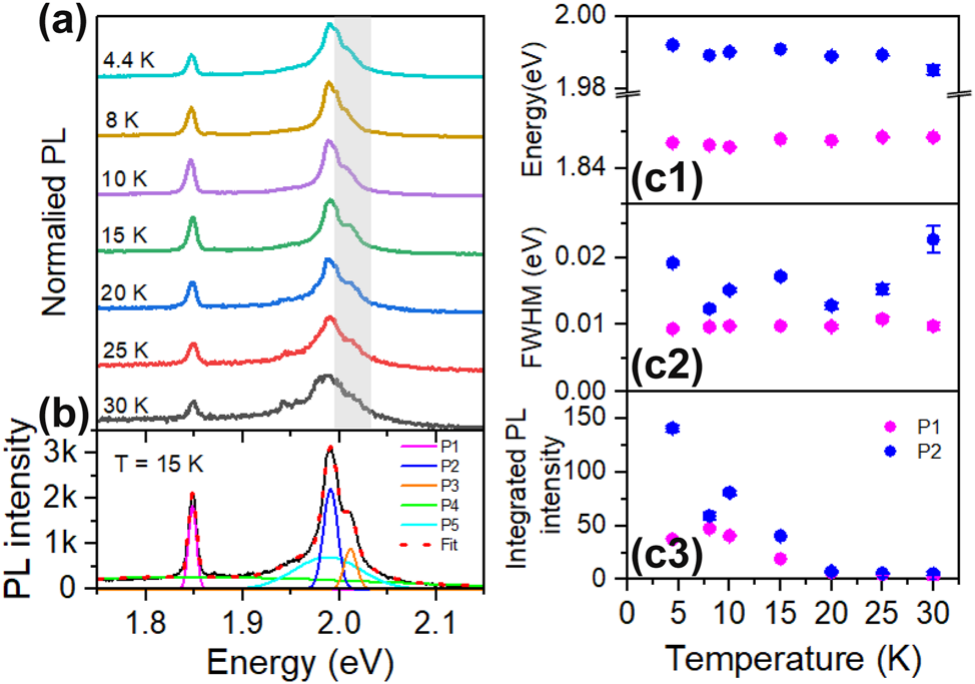
Temperature dependent PL emission. (a) PL spectrum of the same WS_2_ nanoribbon as in Figure 4, measured at various temperatures. (b) PL spectrum at *T* = 15 K along with the fit using multiple Gaussian functions. Temperature dependence of the peak position, full-width at half maximum (FWHM), integrated PL intensity for (c1)–(c3) sharp emission peaks, P1 and P2.

To better understand the origin of different emission components in Figure 4, we also conducted temperature dependent photoluminescence measurements. PL spectra measured at different temperatures are shown in Figure 5(a) with intensity offsets for clarity. The higher energy P3 peak in Figure 4 becomes clearer with increasing temperature, as indicated by the gray shadowed region. We adopt the same fitting method used in Figure 4 to analyze the spectra, with an exemplary fitting result for the *T* = 15 K spectrum shown in Figure 5(b). The temperature dependence of the energy position, width and integrated PL intensity for sharp peaks, P1 and P2, can be seen in Figure 5(c1)–(c3). The P1 peak blueshifts slightly by 1.6 meV, while P2 redshifts by 6.8 meV as the temperature rises from 4.4 K to 30 K. The width of P1 and P2 does not change obviously in the current temperature range as shown in Figure 5(c2), and they remain around 10 meV and 15 meV, respectively. The integrated intensities of both P1 and P2 increase as the sample temperature decrease, which is consistent with the behavior of localized excitons. At higher temperatures, the integrated PL intensity for all components decline, but the sharp peaks still survive up to 30 K. The temperature dependent parameters for P3–P5 can be found in Figure S5.

Based on the analysis in Figures 4 and 5, we can assign P2 to trion emission, P3 to neutral exciton emission, consistent with previous results in the literature [59], [60]. The peak P4 overlaps well with the bilayer emission band as shown in Figure 2, thus we attribute it to bilayer emission. While for P5, its emission wavelength covers both exciton and trion emission ranges, but is much broader, so it possibly results from phonon-assisted exciton or trion emissions.

Despite the reported single photon emission from TMDC monolayers, we haven’t been able to identify the single photon emitter in our nanoribbons. As shown in Figure S8, the second-order photon-autocorrelation for sharp emission lines was measured, but it did not reveal antibunching. This is either due to the influence of ‘strong background’ emissions, which is reflected in the broad Gaussian background, or due to the fact that emissions from nanoribbons can be largely influenced by the local strain and dielectric environment, leading to many more radiative, nonradiative decay processes that perturbs the characteristics of the defect trap potential. Indeed, the observed linewidth of PL emissions from WS_2_ NRs are still one order of magnitude wider than typical TMDC single photon emitters [13], [14], [15], [16]. Further improvement may needs optimization of the fabrication technique, and effective isolation of the nanoribbons from the environment such as *h*-BN encapsulation.

## 4 Discussion

Defect emissions have been commonly observed in monolayer TMDCs, which display spectra of different shapes, e.g. sharp lines or broad bands, due to the varying origins and dielectric environments [20]. Broad low emission peaks similar to those of Figure 2(c) and (d) have been reported in PL studies of bilayer WS_2_ [61] as well as monolayer WS_2_ after plasma treatments where lots of defects are created intentionally [62]. Because our samples have not been subject to any of the defect generation processes, we attributed the broad low energy emission peak of Figure 2(c) and (d) to emission of bilayer WS_2_ NRs, which are produced together with Monolayer NRs in the exfoliation from the slanted crystal edge.

Defect-induced sharp peak emissions have also been reported. For instance, by using He-ion beam bombardment, Micevic et al. create a very sharp peak emission at 1.556 eV in *h*BN/WS_2_/*h*BN heterostructures cooled to 10 K [19]. The same method is applied to create single photon emitters in MoS_2_ ML [17], [63], [64], which are supposed to be related to unpassivated sulfur vacancies. As for our case, the sharp emission lines with energy red-shifted far away from exciton peak could originate from sulfur vacancies, however sulfur vacancy defect-induced emission cannot cover the sharp peaks which are very close to exciton energy [19], [65], [66]. Another candidate mechanism for the near-bandgap sharp peaks could involve localized excitons.

Exciton localization can be categorized by, i.e. strain localization [20], [24] and defect localization [13], [14], [16]. Cianci et al. investigated *h*BN-Capped WS_2_ domes and observed localized exciton induced single photon emission with energy positions very close to free excitons. A similar situation was also observed by Palacios-Berraquero et al. [24] in strained monolayer WS_2_ using silica nanopillars. Obviously, in both cases, it is strain that is mostly responsible for the sharp emission lines. While another work from Palacios-Berraquero et al. [18], reported electrically driven sharp emission from localized sites in ML WS_2_. At 10 K, they observed bright spots in the ML WS_2_ under electrical driving with emission energy around 1.937 eV which also shows the single-photon emission feature. However, because in their work, no nanostructures are used to intentionally create strain, it is hard to determine which type of exciton localization it is, since defects are very common in crystals and strain can form naturally during the sample preparation of atomically thick layers of TMDCs [59], [67]. We encounter the same problem in our case. In order to try to tackle this question, we also perform atomic force microscopy (AFM) measurements as shown in Figure S9 on a sample area where many sharp emission lines are observed, to have more understanding about our sample surface. Many bright spots with higher height are observed, however, these bright spots are contaminants sitting on the top of the nanoribbons [58], which cannot produce strain as nanodomes or nanopillars In Refs. [20], [24]. However, during the sample preparation process, tearing of the ribbons from the vdW bulk crystals may lead to the creation of strain along the edges and throughout the entire ribbon, as is seen in the Raman and SHG spectroscopy measurements [58]. The local strained area may behave as effective traps for charge carriers, leading to the sharp emission lines in the optical emission spectra.

## 5 Conclusions

In this work, we studied the optical properties of TMDC NRs at cryogenic temperatures. We identified multiple emission features for TMDC NRs of monolayer or bilayer area. The PL signature of the ribbons under cryogenic temperatures serves as clear indication of the local strain, defects, and the trapping of the exciton or trion species. The 1D geometry of NRs renders them a unique nano-platform which is more sensitive to such effects, opening up potential possibilities as building blocks in future sensor applications.

## 6 Experimental methods

### 6.1 Micro-photoluminescence spectroscopy

A continuous-wave solid state green laser with energy of 2.33 eV is used for sample excitation, a pulsed blue laser with an energy of 3.06 eV is also used specially in *g* ^2^(*τ*) measurements. The green laser is reflected through a dichroic beam splitter, and the blue one is reflected through a 50:50 beam splitter. Both of them are then focused onto the sample (placed in Oxford Microstat Hires liquid Helium flow cryostat) to a diffraction limited spot size using a 50×, 0.7 NA Olympus objective microscope, which is used to both excite the sample and collect the PL. A lens is put before the objective in the excitation path to expand the laser spot for wide-field measurements, and simply removing the lens will change the setup to the confocal mode. Collected PL after passing through the dichroic beam splitter or 50:50 beam splitter and long-pass filters goes either to a spectrometer with 150 g/mm grating, finally detected either by LN-cooled CCD camera (Acton SP2300i) for PL measurements, or to two avalanche photodiode (APD) in *g* ^2^(*τ*) measurements.

## Supplementary Material

Supplementary Material Details

## References

[1] Wang G. (2018). Colloquium: excitons in atomically thin transition metal dichalcogenides. *Rev. Mod. Phys.*.

[2] Wilson N. P., Yao W., Shan J., Xu X. (2021). Excitons and emergent quantum phenomena in stacked 2D semiconductors. *Nature*.

[3] Xu X., Yao W., Xiao D., Heinz T. F. (2014). Spin and pseudospins in layered transition metal dichalcogenides. *Nat. Phys.*.

[4] Yu H., Cui X., Xu X., Yao W. (2015). Valley excitons in two-dimensional semiconductors. *Natl. Sci. Rev.*.

[5] Atatüre M., Englund D., Vamivakas N., Lee S., Wrachtrup J. (2018). Material platforms for spin based photonic quantum technologies. *Nat. Rev. Mater.*.

[6] Liu X., Hersam M. C. (2019). 2D materials for quantum information science. *Nat. Rev. Mater.*.

[7] Dastidar M. G., Thekkooden I., Nayak P. K., Bhallamudi V. P. (2022). Quantum emitters and detectors based on 2D van der waals materials. *Nanoscale*.

[8] Turunen M. (2022). Quantum photonics with layered 2D materials. *Nat. Rev. Phys.*.

[9] Alivisatos A. P. (1996). Semiconductor clusters, nanocrystals, and quantum dots. *science*.

[10] Hu J., Odom T. W., Lieber C. M. (1999). Chemistry and physics in one dimension: synthesis and properties of nanowires and nanotubes. *Acc. Chem. Res.*.

[11] Montblanch A. R., Barbone M., Aharonovich I., Atatüre M., Ferrari A. C. (2023). Layered materials as a platform for quantum technologies. *Nat. Nanotechnol.*.

[12] Chakraborty C., Kinnischtzke L., Goodfellow K. M., Beams R., Vamivakas A. N. (2015). Voltage controlled quantum light from an atomically thin semiconductor. *Nat. Nanotechnol.*.

[13] He Y. (2015). Single quantum emitters in monolayer semiconductors. *Nat. Nanotechnol.*.

[14] Koperski M. (2015). Single photon emitters in exfoliated WSe_2_ structures. *Nat. Nanotechnol.*.

[15] Srivastava A., Sidler M., Allain A. V., Lembke D. S., Kis A., Imamoglu A. (2015). Optically active quantum dots in monolayer WSe_2_. *Nat. Nanotechnol.*.

[16] Tonndorf P. (2015). Single-photon emission from localized excitons in an atomically thin semiconductor. *Optica*.

[17] Mitterreiter E. (2021). The role of chalcogen vacancies for atomic defect emission in MoS_2_. *Nat. Commun.*.

[18] Palacios-Berraquero C. (2016). Atomically-thin quantum light emitting diodes. *Nat. Commun.*.

[19] Micevic A. (2022). On-demand generation of optically active defects in monolayer WS_2_ by a focused helium ion beam. *Appl. Phys. Lett.*.

[20] Cianci S. (2023). Spatially controlled single photon emitters in *h*BN-capped WS_2_ domes. *Adv. Opt. Mater.*.

[21] Zhao H., Pettes M. T., Zheng Y., Htoon H. (2021). Site-controlled telecom-wavelength single-photon emitters in atomically-thin MoTe_2_. *Nat. Commun.*.

[22] Zhao H. (2023). Manipulating interlayer excitons for near-infrared quantum light generation. *Nano Lett.*.

[23] Li X. (2023). Proximity-induced chiral quantum light generation in strain-engineered WSe_2_/NiPS_3_ heterostructures. *Nat. Mater.*.

[24] Palacios-Berraquero C. (2017). Large-scale quantum-emitter arrays in atomically thin semiconductors. *Nat. Commun.*.

[25] Branny A., Kumar S., Proux R., Gerardot B. D. (2017). Deterministic strain-induced arrays of quantum emitters in a two-dimensional semiconductor. *Nat. Commun.*.

[26] Rosenberger M. R. (2019). Quantum calligraphy: writing single-photon emitters in a two-dimensional materials platform. *ACS Nano*.

[27] Klein J. (2019). Site-selectively generated photon emitters in monolayer MoS_2_ via local helium ion irradiation. *Nat. Commun.*.

[28] Hötger A. (2021). Gate-switchable arrays of quantum light emitters in contacted monolayer MoS_2_ van der waals heterodevices. *Nano Lett.*.

[29] Parto K., Azzam S. I., Banerjee K., Moody G. (2021). Defect and strain engineering of monolayer WSe_2_ enables site-controlled single-photon emission up to 150 K. *Nat. Commun.*.

[30] Stevens C. E. (2022). Enhancing the purity of deterministically placed quantum emitters in monolayer WSe_2_. *ACS Nano*.

[31] Paralikis A. (2024). Tailoring polarization in WSe_2_ quantum emitters through deterministic strain engineering. ..

[32] O’brien J. L. (2007). Optical quantum computing. *Science*.

[33] Couteau C. (2023). Applications of single photons to quantum communication and computing. *Nat. Rev. Phys.*.

[34] Fang M., Yang E. (2023). Advances in two-dimensional magnetic semiconductors via substitutional doping of transition metal dichalcogenides. *Materials*.

[35] Li Y., Zhou Z., Zhang S., Chen Z. (2008). MoS_2_ nanoribbons: high stability and unusual electronic and magnetic properties. *J. Am. Chem. Soc.*.

[36] Botello-Méndez A. R., Lopez-Urias F., Terrones M., Terrones H. (2009). Metallic and ferromagnetic edges in molybdenum disulfide nanoribbons. *Nanotechnology*.

[37] Pan H., Zhang Y. (2012). Edge-dependent structural, electronic and magnetic properties of MoS_2_ nanoribbons. *J. Mater. Chem.*.

[38] López-Urías F., Elias A. L., Perea-Lopez N., Gutierrez H. R., Terrones M., Terrones H. (2014). Electronic, magnetic, optical, and edge-reactivity properties of semiconducting and metallic WS_2_ nanoribbons. *2D Materials*.

[39] Xiao S., Yu W., Gao S. (2016). Edge preference and band gap characters of MoS_2_ and WS_2_ nanoribbons. *Surf. Sci.*.

[40] Pan H., Zhang Y. (2012). Tuning the electronic and magnetic properties of MoS_2_ nanoribbons by strain engineering. *J. Phys. Chem. C*.

[41] Lu P., Wu X., Guo W., Zeng X. (2012). Strain-dependent electronic and magnetic properties of MoS_2_ monolayer, bilayer, nanoribbons and nanotubes. *Phys. Chem. Chem. Phys.*.

[42] Zhang H., Li X., Liu L. (2013). Tunable electronic and magnetic properties of WS_2_ nanoribbons. *J. Appl. Phys.*.

[43] Liu H. (2023). Local strain engineering in Janus MoSSe nanoribbons induces tunable electronic structures and remarkable magnetic moments. *J. Phys. D: Appl. Phys.*.

[44] Yue Q. (2012). Bandgap tuning in armchair MoS_2_ nanoribbon. *J. Phys.:Condens. Matter*.

[45] Chen Y., Lee C., Cheng S., Yang C. (2020). Electronic structures of WS_2_ armchair nanoribbons doped with transition metals. *Sci. Rep.*.

[46] Ataca C., Sahin H., Akturk E., Ciraci S. (2011). Mechanical and electronic properties of MoS_2_ nanoribbons and their defects. *J. Phys. Chem. C*.

[47] Neupane S., Tang H., Ruzsinszky A. (2023). Defect-induced states, defect-induced phase transition, and excitonic states in bent tungsten disulfide (WS_2_) nanoribbons: density functional vs. many body theory. *Phys. Rev. Mater.*.

[48] Fan D. D., Liu H. J., Cheng L., Jiang P. H., Shi J., Tang X. F. (2014). MoS_2_ nanoribbons as promising thermoelectric materials. *Appl. Phys. Lett.*.

[49] Chen K., Luo Z., Mo D., Lyu S. (2016). WSe_2_ nanoribbons: new high-performance thermoelectric materials. *Phys. Chem. Chem. Phys.*.

[50] Wang J. (2017). Excellent thermoelectric properties in monolayer WSe_2_ nanoribbons due to ultralow phonon thermal conductivity. *Sci. Rep.*.

[51] Han D., Wang M., Yang X., Du M., Cheng L., Wang X. (2022). Discovery of high thermoelectric performance of WS_2_-WSe_2_ nanoribbons with superlattice and Janus structures. *J. Alloys Compd.*.

[52] Pandey N., Kumar A., Chakrabarti S. (2019). First principle study of temperature-dependent magnetoresistance and spin filtration effect in WS_2_ nanoribbon. *ACS Appl. Mater. Interfaces*.

[53] Correa J. H., Dias A. C., Villegas-Lelovsky L., Fu J., Chico L., Qu F. (2020). Anisotropy of the spin-polarized edge current in monolayer transition metal dichalcogenide zigzag nanoribbons. *Phys. Rev. B*.

[54] Tang H., Neupane S., Yin L., Breslin J. M., Ruzsinszky A. (2023). Spin-polarization anisotropy controlled by bending in tungsten diselenide nanoribbons and tunable excitonic states. *J. Mater. Chem. C*.

[55] Tang H., Breslin J. M., Yin L., Ruzsinszky A. (2023). Bending effects and optical properties of WSe_2_ nanoribbons of topological phase. *Phys. Rev. Mater.*.

[56] Tang H., Neupane B., Neupane S., Ruan S., Nepal N. K., Ruzsinszky A. (2022). Tunable band gaps and optical absorption properties of bent MoS_2_ nanoribbons. *Sci. Rep.*.

[57] Chowdhury T., Sadler E. C., Kempa T. J. (2020). Progress and prospects in transition-metal dichalcogenide research beyond 2D. *Chem. Rev.*.

[58] Saunders A. P. (2024). Direct exfoliation of nanoribbons from bulk van der waals crystals. *Small*.

[59] Mitioglu A. A. (2013). Optical manipulation of the exciton charge state in single-layer tungsten disulfide. *Phys. Rev. B*.

[60] Shang J. (2015). Observation of excitonic fine structure in a 2D transitionmetal dichalcogenide semiconductor. *ACS Nano*.

[61] Long Y., Huang L. (2015). Exciton dynamics and annihilation in WS_2_ 2D semiconductors. *Nanoscale*.

[62] Chow P. K. (2015). Defect-induced photoluminescence in monolayer semiconducting transition metal dichalcogenides. *ACS Nano*.

[63] Barthelmi K. (2020). Atomistic defects as single-photon emitters in atomically thin MoS_2_. *Appl. Phys. Lett.*.

[64] Klein J. (2021). Engineering the luminescence and generation of individual defect emitters in atomically thin MoS_2_. *ACS Photonics*.

[65] Refaely-Abramson S., Qiu D. Y., Louie S. G., Neaton J. B. (2018). Defect-induced modification of low-lying excitons and valley selectivity in monolayer transition metal dichalcogenides. *Phys. Rev. Lett.*.

[66] Schuler B. (2019). Large spin-orbit splitting of deep ingap defect states of engineered sulfur vacancies in monolayer WS_2_. *Phys. Rev. Lett.*.

[67] Gutiérrez H. R. (2013). Extraordinary room-temperature photoluminescence in triangular WS_2_ monolayers. *Nano Lett.*.

