# Supplementary material for: Localized exciton emission from monolayer WS2 nanoribbon at cryogenic temperature
Source: Nanophotonics. 2025 Jan 7;14(11):1721–8. doi: 10.1515/nanoph-2024-0583 (PMC12133312; doi:10.1515/nanoph-2024-0583)
Supplement: Supplementary file 1 — Supplementary Material Details [file j_nanoph-2024-0583_suppl_001.docx]

**Supplementary materials**

**Localized exciton emission from monolayer WS_2_ nanoribbon at cryogenic temperature**

**Gang Qiang^1^, Ashley P. Saunders^2^, Cong Tai Trinh^1^, Na Liu^1^, Andrew C. Jones^1^, Fang Liu^2^ and Han Htoon^1^***

**^1^** **Center for Integrated Nanotechnologies, Materials Physics and Applications Division,**

**Los Alamos National Laboratory, Los Alamos, NM 87545, USA**

**^2^Department of Chemistry, Stanford University, Stanford, CA 94305, USA**


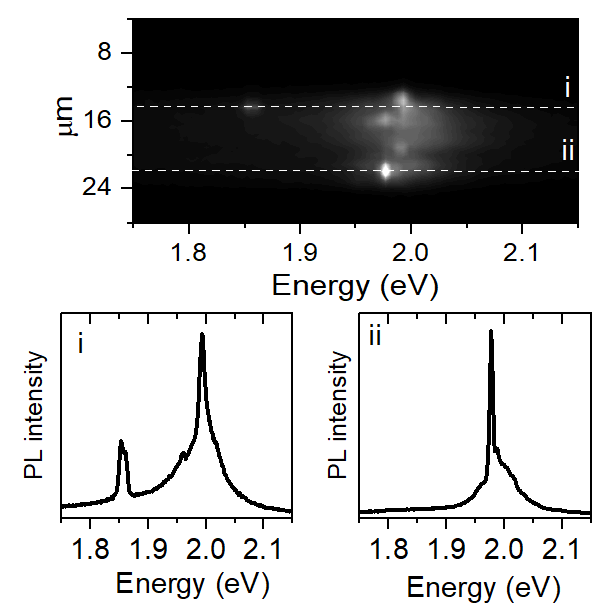


**Fig. S1.** Top: PL signal dispersed on CCD chip from a selected WS_2_ NRs for Fig. 4 and Fig. 5 in the main text. Bottom right: averaged PL spectrum selected from pixel position i (PL34), the corresponding laser excitation power and temperature dependent results are shown in Fig. 4 and Fig. 5. Bottom left, averaged PL spectrum from pixel position ii (PL52).


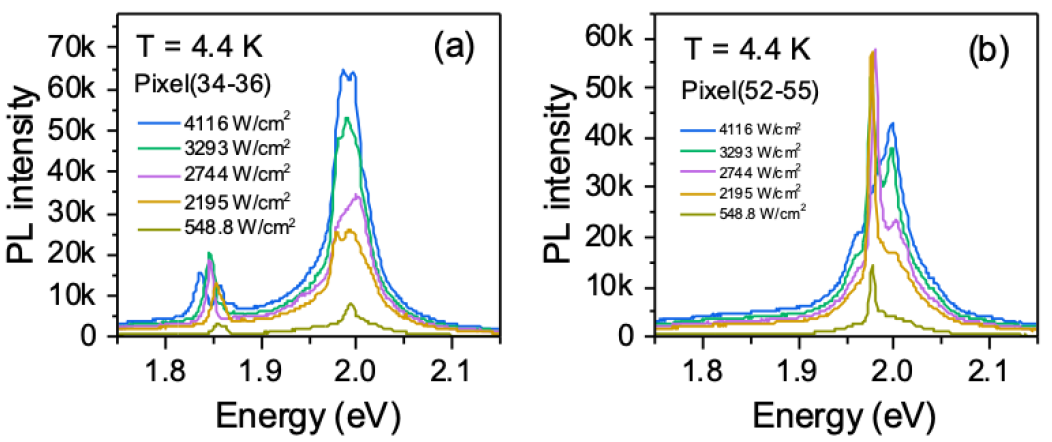


**Fig. S2.** PL34 and PL52 in Fig. S1 under high excitation power at *T* = 4.4 K.


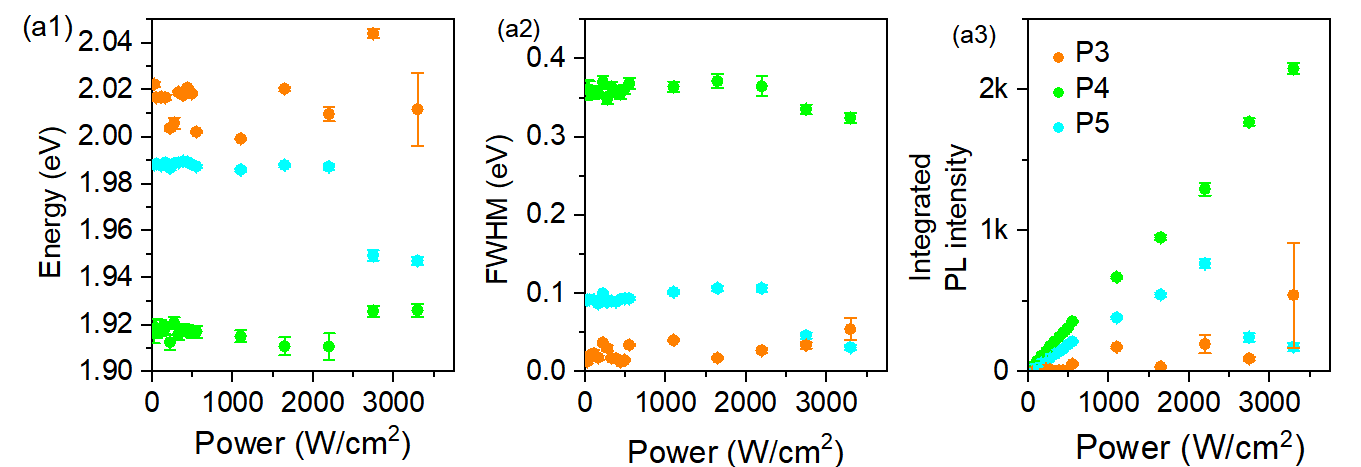


**Fig. S3.** Excitation power dependence of the (a1) peak position, (a2) full-width at half maximum (FWHM), (a3) integrated PL intensity for broad components P3, P4, P5 in Fig. 4.


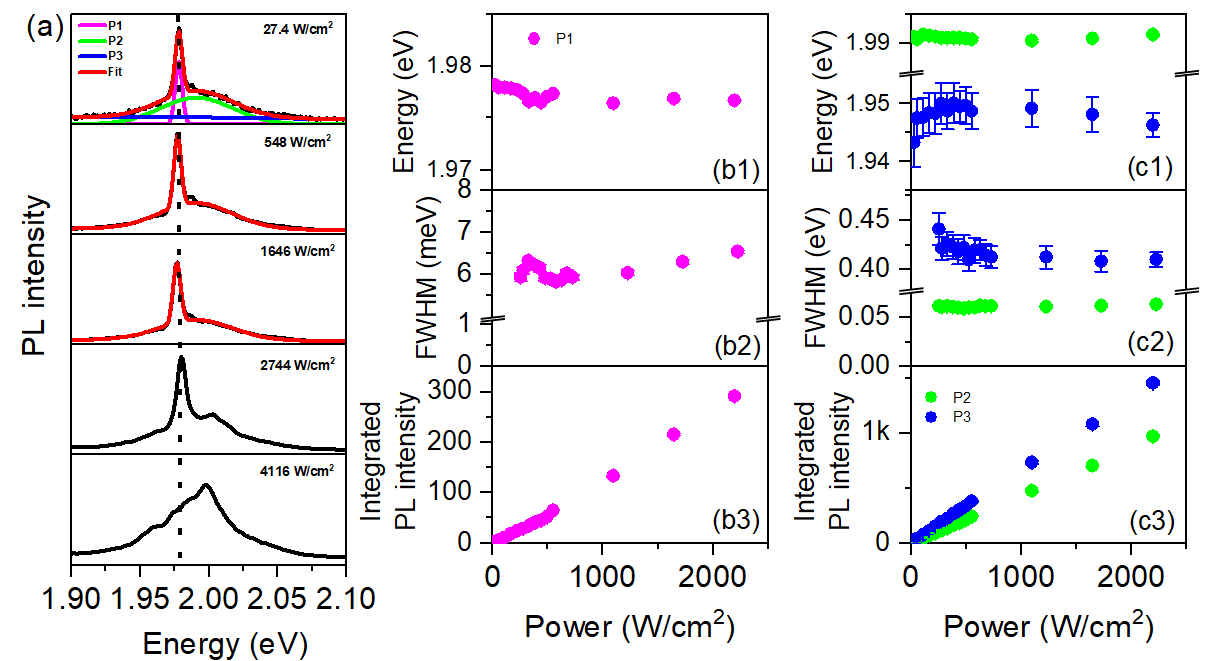


**Fig. S4.** (a) Excitation power dependent PL52 in Fig. S1. The red lines are fits. In the top panel, all Gaussian fitting components are shown. Dashed vertical black line is guide for eyes. Excitation power dependence of the peak position, full-width at half maximum (FWHM), integrated PL intensity (b1)-(b3) for sharp emission peak P1 and (c1)-(c3) for broad peaks P2 and P3. Experiments are done at *T* = 4.4 K.

Figure S4(a) displays PL52 measured under different excitation powers, to analyze the data, we fit each spectrum with three Gaussian functions. However, under very high laser excitation power, the shape of PL spectra change a lot as indicated in Figure S2(b), therefore we only focus on the analysis of PL spectra whose shape are not changed by the higher excitation power. Red curves in Fig. S4(a) are fits, in the top panel, the Gaussian components for PL spectrum are also shown. The power dependence of the extracted energy, width and integrated PL intensity for sharp peaks (P1) are shown in Fig. S4(b1)-(b3) and those for broad peaks (P2 and P3) are shown in Fig. S4(c1)-(c3). With the increasing of laser excitation power, the energy position of sharp peak P1 (magenta) and broad peak P2 (green) red-shift slightly at the beginning, then P1 peak energy remain almost constant, but P2 peak energy tends to increase. In contrast, the energy position of P3 (blue) increases firstly and turns to decline. The width of P1 and P2 increases with the increasing of excitation power, while for P3, it decreases. The integrated PL intensity for all components (P1-P3) increases almost linearly with excitation power.


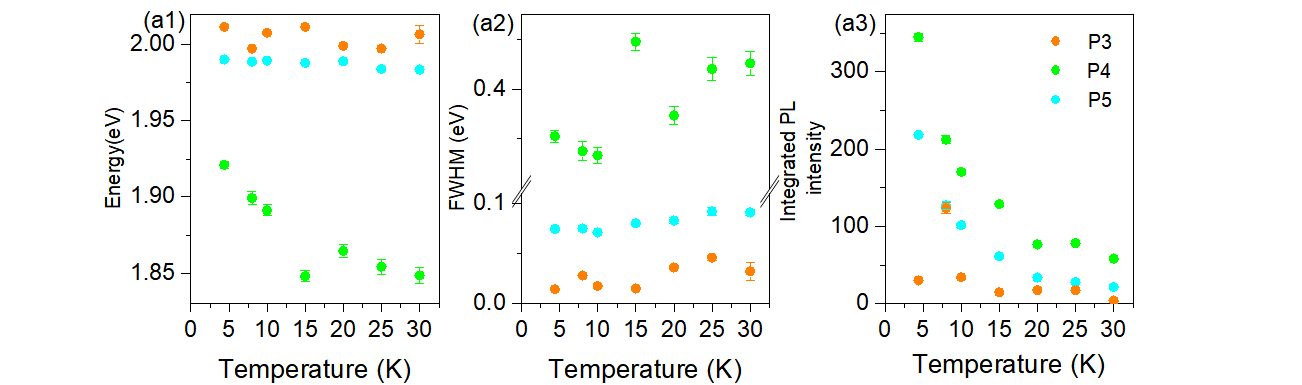


**Fig. S5.** Temperature dependence of the (a1) peak energy, (a2) full-width at half maximum (FWHM), (a3) integrated PL intensity for exciton peak P3 and broad components P4 and P5 in Fig. 5.

The temperature dependence of the energy position, width and integrated PL intensity for exciton peak P3 and broad peaks (P4 and P5) in Fig. 5 are shown in Fig. S5(d1)-(d3). With the rise of temperature from 4.4 K to 30 K, P3 peak energy fluctuates around 2.005 eV. While P4 redshifts clearly by 73 meV from 1.921 eV to 1.848 eV, but P5 only redshifts about 6.7 meV. The width of P3-P5, increases slightly in general, and the integrated PL intensity for all components declines at higher temperature.


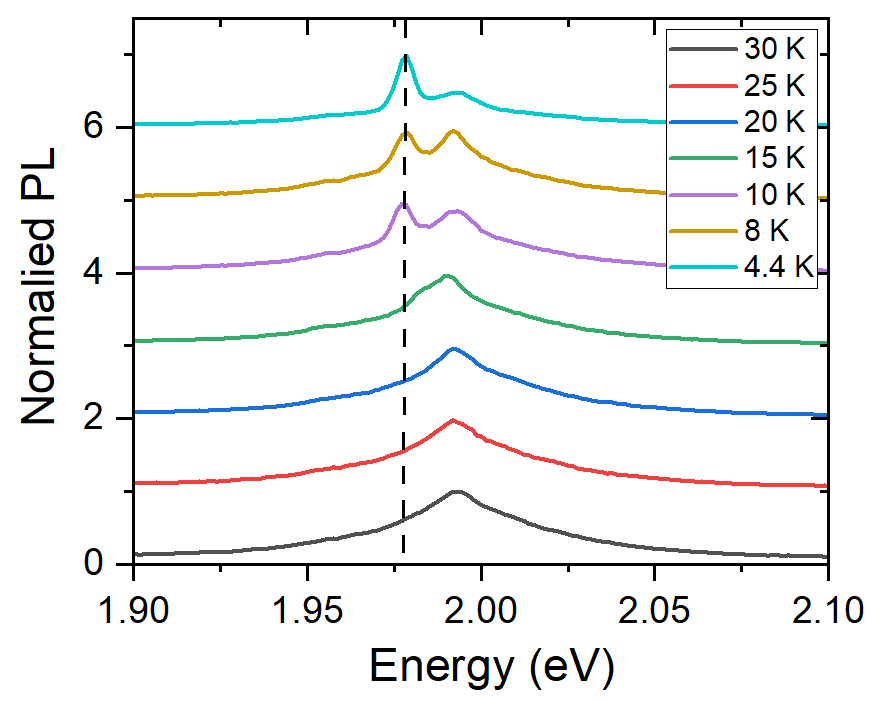


**Fig. S6.** Temperature dependent PL52 in Fig. S1.

**
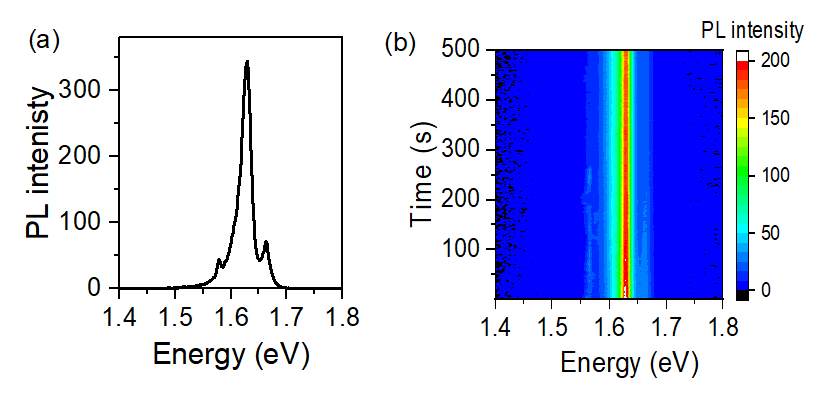
**

**Fig. S7.** (a) PL spectrum of MoSe_2_ NRs, and (b) it time stability. Measurements are done at *T* = 4.4 K.


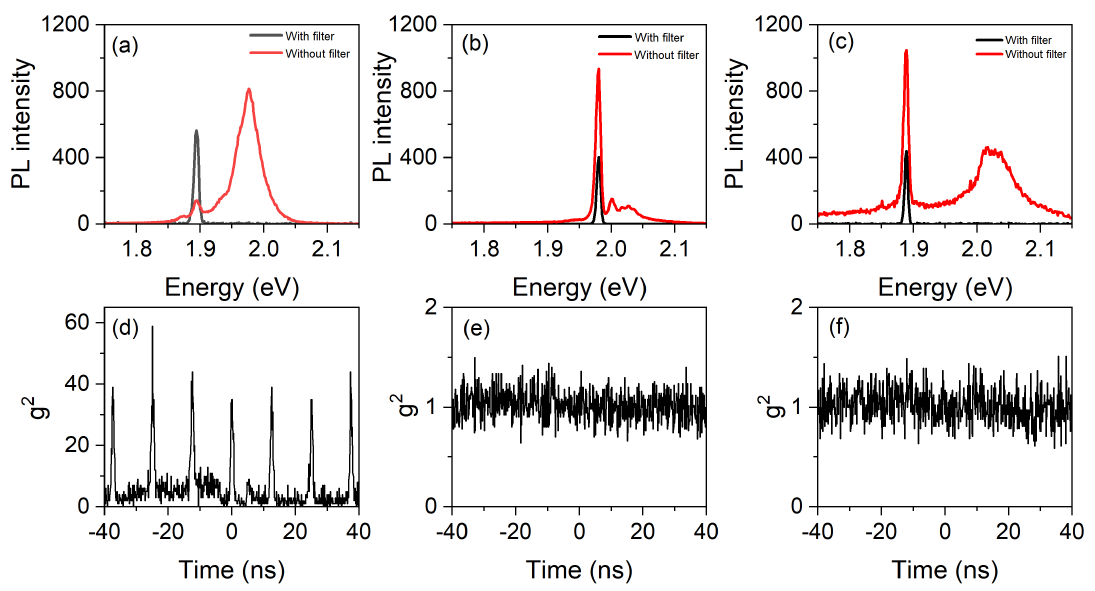


**Fig. S8.** Second-order photon-autocorrelation (g^2^(τ)) measurements.(a)-(b) PL spectra measured under confocal mode at three different sample points. Red lines are spectra measured without filter, black lines are spectra measured with filter to pick out the sharp emission line for g^2^(τ) measurements as shown in (d)-(f). g^2^(τ) measured (d) under 440 pulsed laser excitation, (e)-(f) under 532 continuous-wavelength laser excitation. All the measurements are done at *T* = 4.4 K.


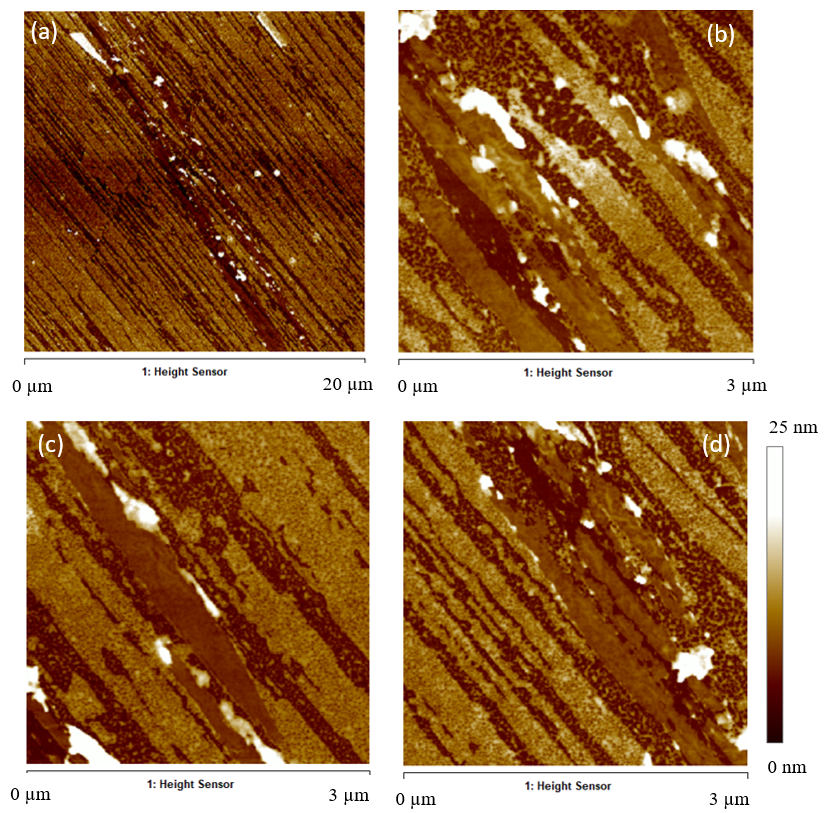


**Fig. S9.** AFM topography of WS_2_ NRs on SiO_2_/Si substrate in an area of (a) 20 μm x 20 μm, (b)-(d) 3 μm x 3 μm.
